# Development of PLA/Lignin Bio-Composites Compatibilized by Ethylene Glycol Diglycidyl Ether and Poly (ethylene glycol) Diglycidyl Ether

**DOI:** 10.3390/polym15204049

**Published:** 2023-10-11

**Authors:** Abdul Shakoor Shar, Ningning Wang, Tianyu Chen, Xiaoying Zhao, Yunxuan Weng

**Affiliations:** 1 College of Chemistry and Materials Engineering, Beijing Technology and Business University, Beijing 100048, China; ashakoorshar1234@outlook.com (A.S.S.); 15624987408@163.com (N.W.); 2130042044@st.btbu.edu.cn (T.C.); 2 Beijing Key Laboratory of Quality Evaluation Technology for Hygiene and Safety of Plastics, Beijing 100048, China

**Keywords:** poly (lactic acid), lignin, ethylene glycol diglycidyl ether, barrier performance

## Abstract

Poly (lactic acid) (PLA) is a promising green substitute for conventional petroleum-based plastics in a variety of applications. However, the wide application of PLA is still limited by its disadvantages, such as slow crystallization rate, inadequate gas barrier, thermal degradation, etc. In this study, lignin (1, 3, 5 PHR) was incorporated into PLA to improve the thermal, mechanical, and barrier properties of PLA. Two low-viscosity epoxy resins, ethylene glycol diglycidyl ether (EGDE) and poly (ethylene glycol) diglycidyl ether (PEGDE), were used as compatibilizers to enhance the performance of the composites. The addition of lignin improved the onset degradation temperature of PLA by up to 15 °C, increased PLA crystallinity, improved PLA tensile strength by approximately 15%, and improved PLA oxygen barrier by up to 58.3%. The addition of EGDE and PEGDE both decreased the glass transition, crystallization, and melting temperatures of the PLA/lignin composites, suggesting their compatabilizing and plasticizing effects, which contributed to improved oxygen barrier properties of the PLA/lignin composites. The developed PLA/lignin composites with improved thermal, mechanical, and gas barrier properties can potentially be used for green packaging applications.

## 1. Introduction

The evolution of plastic pollution is a global concern [1]. Plastic, with its durable and versatile nature, has revolutionized various industries, offering convenience and affordability. However, its inability to degrade naturally presents a significant challenge. Awareness of the detrimental effects of plastic pollution has grown steadily, and efforts to reduce plastic consumption, promote plastic recycling, and develop sustainable alternatives have gained increasing attention [2].

Bioplastics are considered a green replacement for traditional plastics due to their renewability, biodegradability, and acceptable mechanical and thermal properties [3,4]. The adoption of biopolymers in the packaging industry represents a crucial stride toward a more sustainable and environmentally responsible future. The significance of using biopolymers lies in their ability to mitigate the detrimental environmental impacts associated with traditional petroleum-based plastics. Scientifically, biopolymers are derived from renewable resources such as plants, starches, and microorganisms, offering a carbon-neutral alternative to fossil fuels [5]. Their production typically requires fewer greenhouse gas emissions, reducing the overall carbon footprint of packaging materials. Moreover, biopolymers exhibit impressive biodegradability and composability, ensuring that packaging waste does not persist in the environment for extended periods. As our understanding of these materials continues to advance, biopolymers stand as a testament to the packaging industry’s commitment to sustainable practices, offering a scientific foundation for eco-conscious packaging solutions that align with the pressing need for a cleaner, greener planet [6]. Bio-polymers have found a multitude of applications in the food packaging industry [7], offering a range of benefits that align with both sustainability and food safety requirements. One key advantage is their biodegradability, which addresses the issue of plastic pollution by ensuring that packaging materials break down naturally over time, reducing environmental harm. Moreover, bio-polymers derived from renewable sources, such as cornstarch and sugarcane, decrease reliance on finite fossil fuels, contributing to a more sustainable supply chain. These materials can also serve as effective barrier coatings, preserving food freshness by preventing moisture and oxygen ingress. Additionally, bio-polymers often exhibit lower levels of chemical migration into food products, enhancing food safety and quality. In summary, the use of bio-polymers in food packaging not only aligns with eco-friendly initiatives but also offers improved protection and safety for packaged foods, making them a vital component of the industry’s sustainable future [8,9].

Polylactic acid (PLA), a bio-based and biodegradable (in defined conditions) polyester [1], is usually formed by polycondensation of lactic acid (LA) or ring-opening polymerization of lactide, a cyclic diester of LA [10,11]. There are two enantiomers of lactic acid: L-lactic acid and D-lactic acid [12]. The incorporation ratio of D-lactic acid and L-lactic acid during polymerization determines the physical properties such as crystallinity, hydrophobicity, and degradation ability of PLA [13,14,15,16]. PLA can be synthesized from both renewable and non-renewable resources [17]. Bio-based PLA is derived from renewable resources such as cornstarch or sugarcane [18,19]. Besides its advantages, there are some inherent properties of PLA that limit its industrial use. PLA has high brittleness, low ductility, slow crystallization rate, poor heat resistance, and inadequate moisture and gas barrier for industrial applications such as packaging [20]. To enhance the properties of PLA for broader applications, a variety of biodegradable and nonbiodegradable materials, such as PLA blend with 10 wt% of chitosan, can improve the thermal and mechanical properties of PLA [21], PLA/PCL blends with a component of 80/20 performs 16 times higher of impact energy compared with pure PLA and exceeds the toughness of pure PCL impact modifier [22]. Arrieta et al. used 15 wt% acetyl tributyl citrate (ATBC) as a plasticizer to plasticize the PLA/PHB blend, which shows an improvement in the thermal stability of the blend up to 9 min [23]. The addition of montmorillonites into provides better dispersion of hybrids clay mineral–graphene (Gr) in the PLA matrix and enhances melt viscosity and storage modulus of Gr–PLA mixtures [24].

Lignin is an abundant and cheap biopolymer comprised of cross-linked, branched aromatic monomers, including p-coumaryl alcohol, coniferyl alcohol, and sinapyl alcohol [25]. Lignin has been introduced into PLA to reduce cost and add new functionalities, such as barrier, heat resistance, and UV shielding properties, without sacrificing the biodegradability and renewability of the PLA matrix [26]. However, blending lignin with PLA is challenging as lignin has high polarity and tends to aggregate in the PLA matrix. As a result, the addition of lignin usually harmed the mechanical performance of PLA due to the inadequate interfacial adhesion and the lignin aggregation [26]. Therefore, lignin is often chemically modified, plasticized, or compatibilized to improve its dispersion and interaction with PLA [27,28,29]. For example, the tensile elongation at break of PLA/lignin composite was improved by 35% with the addition of 2 wt% poly(ethylene glycol) as a plasticizer [30]. Surface modification of lignin via acetylation improved the mechanical properties of PLA/lignin composites at low lignin loading (<5%), while higher lignin loading reduced the mechanical properties [31]. Grafting of PLA onto lignin improved lignin in the PLA matrix at low lignin loading. In addition to the fact that chemical modification of lignin usually works at low lignin loading, it generally involves complex and time-consuming procedures, which is not preferred for industrial applications. Therefore, it is necessary to develop economical and facile techniques to improve the interfacial interaction and mechanical properties of PLA/lignin composites.

Comparing PLA/Lignin bio-composite packaging films with traditional plastic packaging films reveals significant advantages in terms of environmental sustainability and performance characteristics. Firstly, from an ecological standpoint, PLA/Lignin bio-composite films are derived from renewable resources, primarily plant-based materials like cornstarch and lignin, making them biodegradable and reducing reliance on fossil fuels, in contrast to traditional plastic films derived from petrochemicals. This attribute makes PLA/Lignin films a more environmentally responsible choice, contributing to reduced carbon footprint and plastic waste. Additionally, PLA/Lignin films are compostable under specific conditions, further minimizing their environmental impact [32].

Scientifically, PLA/Lignin bio-composite films exhibit notable mechanical and barrier properties. They possess comparable tensile strength and flexibility to traditional plastic films, making them suitable for various packaging applications. Furthermore, PLA/Lignin films demonstrate impressive gas barrier properties, effectively preserving the freshness and shelf life of packaged products. Their gas barrier performance is attributed to the incorporation of lignin, which acts as a natural barrier to oxygen and moisture, surpassing the permeability of many conventional plastic films. In short, the superiority of PLA/Lignin bio-composite packaging films over traditional plastic packaging films is evident via their sustainable sourcing, biodegradability, and enhanced barrier properties. This advancement aligns with the global shift towards eco-friendly materials and the reduction in plastic waste. PLA/Lignin films offer a scientifically supported, environmentally responsible alternative that holds great promise for a more sustainable and greener future in the packaging industry [33,34].

Therefore, the present work investigates the compatibilizing and plasticizing effects of two low-viscosity epoxy resins, ethylene glycol diglycidyl ether (EGDE) and poly (ethylene glycol) diglycidyl ether (PEGDE), on PLA/lignin composites. Epoxy resins are used as compatabilizing agents in food packaging to improve the adhesion and compatibility between different polymer layers. In multi-layer packaging materials, various polymers are often combined to achieve specific properties like barrier protection and strength. Epoxy resins also serve as plasticizing agents in food packaging by enhancing the flexibility and processability of polymer materials. Epoxy resins contain epoxide functional groups (oxirane rings) that have high reactivity with a variety of functional groups found in different polymers. The epoxy groups can chemically bond with the functional groups on the surface of other polymers, creating covalent bonds or hydrogen bonds. This chemical linkage effectively bridges the interface between the different polymer layers, improving their adhesion and compatibility. In the case of EVOH (Ethylene Vinyl Alcohol) and polyethylene, for example, the epoxy resins can form hydrogen bonds with the hydroxyl groups in EVOH, ensuring a strong interfacial bond. The plasticizing effect of epoxy resins arises from their ability to increase the free volume and reduce intermolecular forces within the polymer matrix. This, in turn, lowers the glass transition temperature (Tg) of the polymer, making it more flexible at ambient temperatures. The scientific basis for this mechanism involves the epoxy resin molecules inserting themselves between polymer chains, effectively acting as a lubricant. This reduces the intermolecular forces (Van der Waals forces) between polymer chains, allowing them to move more freely. As a result, the polymer becomes less brittle and more pliable. The epoxy resin’s molecular structure, with its long flexible chains, aids in this process [35,36]. The thermal, barrier, and mechanical properties of the developed PLA/lignin bio-composites were characterized to provide information about their industrial applications.

## 2. Materials and Methods

### 2.1. Materials

**Figure 1:**
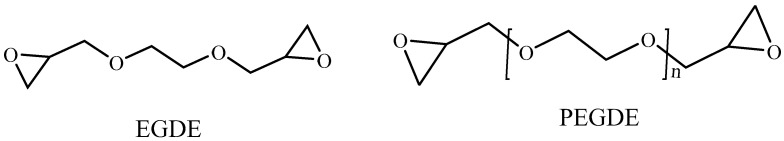
Chemical structure of EGDE and PEGDE.

Poly (lactic acid) (PLA, 4032D) with a specific gravity of 1.24 g/cm^3^ and melting point of 155–170 °C was purchased from NatureWorks LLC, Blair, NE, USA. Lignin (Mn = 2265 Da, chloroform as eluent) was purchased from Shanghai Dongsheng New Material Co., Ltd., Shanghai, China. Both PLA resin and lignin were dried under vacuum at 80 °C for 12 h before use. ethylene glycol diglycidyl ether (EGDE, the epoxy equivalent is 112~135 g/eq) and poly (ethylene glycol) diglycidyl ether (PEGDE, Mn = 485 g/mol) was purchased from Beijing Innochem Technology Co., Ltd., Beijing, China (Figure 1). Both EGDE and PEGDE were of analytical grade and were used as received without further treatment. 

### 2.2. Preparation of PLA/Lignin Bio-Composite Compatibilized with EGDE and PEGDE

PLA/lignin bio-composite compatibilized with EGDE and PEGDE were manufactured using an internal mixer at 180 °C and a rotation speed of 50 r/min for 6 min. The sample formula is shown in Table 1.

### 2.3. Preparation of PLA/Lignin Bio-Composite Film

PLA/lignin bio-composite was hot-pressed into a film using a hydraulic press machine (LP-S-50, Lab-Tech Inc., Beijing, China) at 180 °C and 6.5 MPa. The thickness of the film was 100–160 μm.

### 2.4. Characterization of Bio-Composite Films

Differential scanning calorimetry (DSC) was performed on a DSC machine (Hitachi Instruments 7020, Tokyo, Japan) at a heating rate of 10 °C/min from 40 to 200 °C under nitrogen. The samples were tested with two consecutive scans. The glass transition (Tg) and melting temperature (Tm) of each sample were determined based on the mid-point transition temperature of the second heating curve. The formula for the crystallinity (χc) calculation is shown as follows:

(1) $$\chi_{c}=\frac{∆Hm-∆Hcc}{w_{f}{∆H}^{0}m}\times 100\%$$

where $\chi_{c}$ is weight fraction crystallinity, ∆Hcc, ∆Hm, and ${∆H}^{0}m$ is the cold crystallization enthalpy, melting enthalpy, and theoretical melting enthalpy of 100% crystalline PLA (${∆H}^{0}m$ = 93.7 J/g), and $w_{f}$ presents the weight fraction of component PLA in composites. 

Wide-angle X-ray diffraction (WAXD) was carried out on WXRD equipment (Rigaku SmartLab, Neu Isenburg, Germany) using Ni-filtered CuK α radiation from 5° to 40° with a scanning rate of 3°/min at room temperature. 

The morphology of the film was characterized using scanning electron microscopy (Quanta 250 FEG, FEI, Hillsboro, OR, USA). The cross-section morphology of the sample films was observed after they were brittlely fractured in liquid nitrogen.

Oxygen (O_2_: purity of 99.99%) transmission rate of the film was tested using an Oxygen Transmission (Rate Tester 31M, Labthink, Jinan, China) at 23 °C and a relative humidity was 30% according to ASTM D1413 [37].

The water vapor barrier of the film was tested using a Moisture Tester (PERMATRAN-W3/33, MOCON, Shanghai, China) according to ISO 15106-3 [38].

The tensile properties of the film were tested using a Zwick/Roell universal testing machine (Zwick/Roell, Guangzhou, China) at room temperature at a crosshead speed of 3 mm/min. At least five specimens were tested for each batch to obtain the mean value.

## 3. Results

### 3.1. TGA

The thermal degradation behavior of PLA/lignin composites plays a critical role in determining their suitability for various applications within the packaging industry. In the study, it was observed that all samples exhibited a single thermal degradation stage, indicating a degree of miscibility between PLA and lignin fillers, as indicated in Table 2. The onset (To) and peak (Tp) degradation temperatures of pure PLA were found to be 340.2 °C and 370.4 °C, respectively (Table 2), serving as reference values for comparison. Interestingly, without the presence of a compatibilizer, the addition of lignin at lower loadings (1 and 3 PHR) led to a noteworthy increase in the onset degradation temperature of PLA, with a rise of approximately 11.4–14.9 °C. However, when lignin was added at a higher loading (5 PHR), a slight decrease in the onset temperature (To) of PLA by approximately 10 °C was observed. The peak degradation temperature of the non-compatibilized PLA/lignin composite remained close to that of neat PLA but exhibited a slight decrease with increasing lignin loading. In contrast, the introduction of EGDE compatibilizer had a favorable effect on the thermal degradation behavior of the PLA/lignin composite. With EGDE, the addition of lignin increased the onset degradation temperature (To) of PLA by 8.6–11.5 °C, while it had no significant effect on the peak degradation temperature (Tp) of PLA. Moreover, the incorporation of PEGDE compatibilizer, especially at lower concentrations (1 and 3 PHR), yielded similar improvements in PLA thermal stability as observed with EGDE. However, at higher PEGDE loading (5 PHR), both the onset and peak degradation temperatures of the PLA/lignin composite were significantly reduced. Overall, the findings indicate that the addition of lignin at 1 and 3 PHR concentrations enhances the thermal stability of PLA, which is particularly advantageous for its thermal processing and practical applications in the packaging industry. The role of compatibilizers like EGDE and PEGDE in optimizing the thermal behavior of PLA/lignin composites underscores their potential to fine-tune material properties, making them more suitable for specific packaging requirements and promoting sustainability in the industry.

### 3.2. DSC

**Figure 2:**
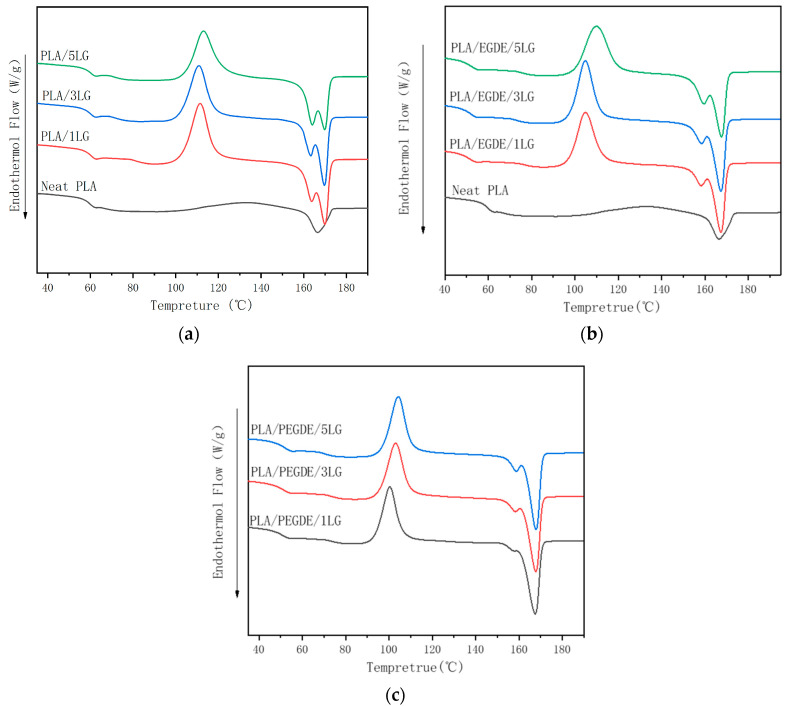
DSC thermograms of (**a**) non-compatibilized PLA/lignin composites; (**b**) EGDE-compatibilized PLA/lignin composites; (**c**) and PEGDE-compatibilized PLA/lignin composites.

Neat PLA had a glass transition at 59 °C, a crystallization peak at 112.8 °C, and a melting peak at 165.6 °C (Figure 2, Table 3). The non-compatibilized PLA/lignin composite had similar glass transition and crystallization temperature as the neat PLA but two melting peaks at approximately 163 and 169 °C, respectively. The formation of the two melting peaks could be caused by melt recrystallization [39]. The crystals formed during the recrystallization process were perfecter than the initial crystals and, therefore, melted at higher temperatures (169 °C). The addition of the lignin increased the crystallinity of PLA. The formation of the two melting peaks could be caused by melt recrystallization [40]. Due to the nucleating effect of the lignin fillers, as we previously reported [41], the lignin loading of 3 PHR has the most significant effect.

The addition of EGDE and PEGDE both decreased the glass transition, crystallization, and melting temperatures of the PLA/lignin composites, suggesting interactions between the PLA matrix and lignin filler and the compatabilizing effect of EGDE and PEGDE. In addition, EGDE and PEGDE can act as a plasticizing agents and cause a decrease in the glass transition temperature of the composites. It is worth mentioning that the melting peaks of the PLA/lignin composites compatibilized by PEGDE were narrower than those of the composites compatibilized by EGDE, suggesting the former had better compatibilizing efficiency.

### 3.3. WAXD

**Figure 3:**
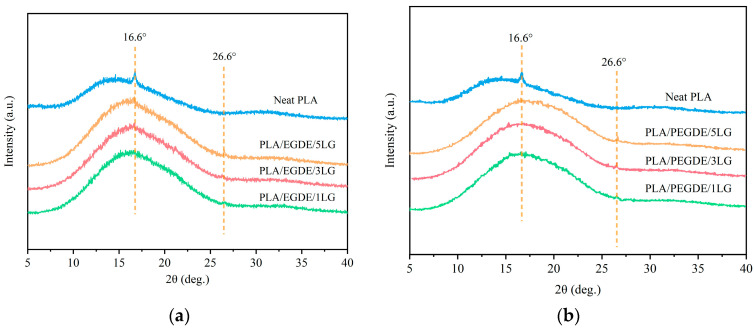
WAXD patterns of neat PLA and the composites (**a**) EGDE-compatibilized PLA/lignin composites; (**b**) and PEGDE-compatibilized PLA/lignin composites.

The crystallization behavior of PLA, alone and in the composites, was investigated using WAXD (Figure 3). Generally, PLA has four crystal forms, which are α, α′, β, and γ [42]. The α crystalline form is the most common one and is obtained from slow cooling of the melt, which allows the molecular chain to rotate into a conformation with lower potential energy; the β crystalline form arises from the deformation of the α crystals and is formed by drawing PLA at elevated temperatures; the γ form is obtained using epitaxial crystallization on a substrate such as hexamethylbenzene [42]. The main characteristic peaks of the α crystal appear at 15°, 17°, 19°, 29°, 31°, and 32°, while the diffraction peaks of β crystals mainly appear at 25°, 26.5°, 27.9°, 29.8°, and 31° [43,44].

A wide diffraction peak from 10° to 25° caused using the scattering of the PLA polymer matrix was observed in the WAXD pattern (Figure 3). The main characteristic diffraction peak of pristine PLA was at 16.6°, corresponding to the (110) and (200) crystal planes of the α crystal form of PLA. The composites of PLA and lignin showed a higher peak intensity at 16.6°, suggesting its higher degree of crystallinity compared with pristine PLA due to the nucleating effect of the lignin particles. A sharp diffraction peak at 26.6° related to an interlayer spacing of 0.34 nm was observed in the composites [45], which was absent in pristine PLA. There are no obvious diffraction peaks at 16.6° on the WAXD patterns of the PLA/lignin composites, indicating a restricted crystallization of PLA in the composites with the presence of the compatibilizers [46,47], which is consistent with the observations from the DSC results.

### 3.4. Tensile Properties

**Figure 4:**
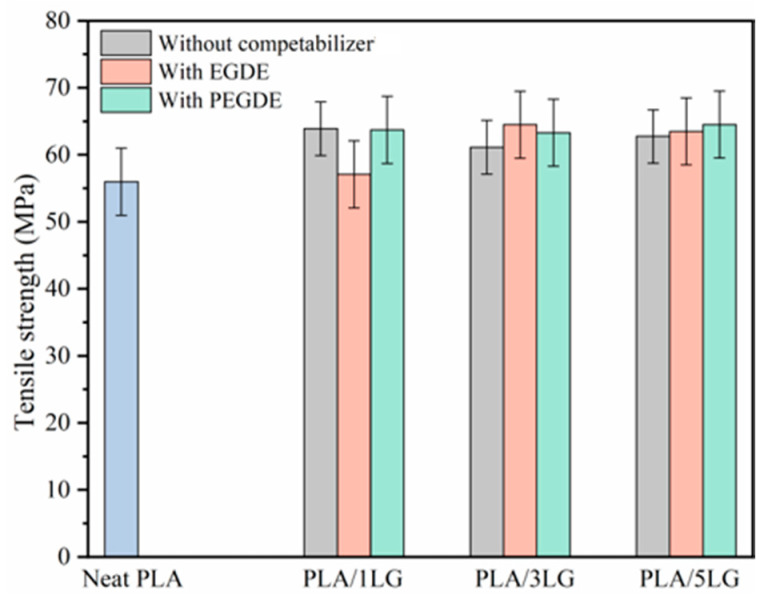
Tensile strength of neat PLA and PLA/lignin compatibilized composite films.

In our examination of mechanical properties, we discovered that pure PLA films boasted a tensile strength of approximately 56 MPa, as illustrated in Figure 4. Interestingly, when lignin was introduced into the mix, even at varying loadings, it resulted in only a modest uptick in PLA’s tensile strength, hovering around a 15% increase. Importantly, this increase did not seem to correlate significantly with the amount of lignin added. Furthermore, the incorporation of EGDE or PEGDE compatibilizers into the PLA/lignin composite did not lead to substantial enhancements in the material’s tensile strength. This outcome surprised us, given that compatibilizers are generally expected to improve material compatibility and, consequently, mechanical properties. This finding stands in contrast to our prior research involving PLA/lignin composites compatibilized with polylactide-graft-glycidyl methacrylate (PLA-g-GMA), where we observed a notable 18.7% [41] increase in tensile strength, particularly when using 3 PHR lignin loading. To unravel this discrepancy and provide a more comprehensive explanation, further investigations into the compatibilization mechanisms of EGDE and PEGDE are needed. These results underscore the intricate nature of polymer interactions and underscore the importance of a detailed analysis to fine-tune material properties for specific applications within the packaging industry.

### 3.5. Oxygen Barrier Performance

The addition of lignin to PLA had a remarkable impact on enhancing the oxygen barrier properties of the composite materials, and what’s particularly interesting is that this improvement was not contingent on the amount of lignin added. In fact, among the compatibilized composites, PLA/1LG exhibited the most exceptional oxygen barrier performance (as detailed in Table 4). It’s worth noting that the effect of lignin loading on oxygen permeability followed a somewhat non-intuitive pattern. Compatibilized PLA/lignin composites with low lignin loadings (e.g., 1 PHR) displayed an increase in oxygen permeability, which essentially means a reduction in the material’s oxygen barrier performance when compared to the compatibilized counterparts. On the other hand, compatibilized composites with higher lignin loadings (specifically, 3 and 5 PHR) demonstrated significant improvements in their oxygen barrier characteristics. Of notable mention, the PLA/EGDE/5LG and PLA/PEGDE/5LG compositions stood out with the most impressive enhancements, exhibiting an astonishing 58.3% and 57.3% improvement in their oxygen barrier properties, respectively. This demonstrates that both EGDE and PEGDE compatibilizers had similar and highly beneficial effects on elevating the oxygen barrier of the PLA/lignin composites. These findings underscore the significant potential of incorporating lignin and compatibilizers like EGDE and PEGDE to tailor the oxygen barrier properties of PLA-based packaging materials. Such advancements hold great promise for extending the shelf life and freshness of packaged food products while also aligning with the industry’s growing emphasis on sustainable packaging solutions. 

The improved gas barrier of the PLA/lignin composites is probably due to the heterogeneous crystal nucleating effects of the lignin particles, which promoted PLA crystallization and caused the rapid formation of small PLA crystals, as supported by the DSC and WAXD results, which functioned as an obstacle to gas diffusion and resulted in improved oxygen barrier [41]. For the compatibilized composites, the bonding and non-bonding of compatibilizer molecules with macromolecular substances in the composites will also affect the dissolution and dispersion of oxygen [48]. The polar groups, such as hydrogen bonds in lignin, increase in number. In addition, due to the high tension of the three-membered ring, the epoxide groups in EGDE or PEGDE are likely to make alcoholic and phenolic compounds in polylactic acid and lignin. The group undergoes a ring-opening reaction to form a certain cross-linked structure. The cross-linked structure has no effect on the solubility of the gas in the polymer, but it will interfere with the formation of the instantaneous fracture gap and decompose the permeability coefficient of the gas. Therefore, the oxygen barrier performance was improved. 

## 4. Conclusions

PLA/lignin composites with the addition of epoxy resins showed that EGDE and PEGDE both decreased the glass transition, crystallization, and melting temperatures of the PLA/lignin composites and improved the oxygen barrier properties of the PLA/lignin composites due to the compatabilizing effect. The addition of lignin improved the onset degradation temperature of PLA by up to 15 °C; the addition of lignin at 1 and 5 PHR showed better results. Addition of lignin 3PHR increased PLA crystallinity, improved PLA tensile strength by approximately 15%, and improved PLA oxygen barrier by up to 58.3%. The PLA/lignin composites with improved properties can potentially be used as green substitutes for conventional petroleum-based plastics in a variety of applications, such as food packaging.

## Figures and Tables

**Table 1:** Composition of PLA/lignin composites.

Sample Codes	PLA (wt%)	Lignin (PHR)	EGDE (PHR)	PEGDE (PHR)
Neat PLA	100	0	0	0
PLA/1LG	100	1	0	0
PLA/3LG	100	2	0	0
PLA/5LG	100	3	0	0
PLA/EGDE/1LG	100	1	5	0
PLA/EGDE/3LG	100	3	5	0
PLA/EGDE/5LG	100	5	5	0
PLA/PEGDE/1LG	100	1	0	5
PLA/PEGDE/3LG	100	3	0	5
PLA/PEGDE/5LG	100	5	0	5

**Table 2:** Thermal degradation temperatures of PLA and PLA/lignin composites.

Sample	To (°C)	Tp (°C)
Neat PLA	340.2	370.4
PLA/1LG	355.1	371.5
PLA/3LG	351.6	369.1
PLA/5LG	330.5	367.5
PLA/EGDE/1LG	348.8	368.4
PLA/EGDE/3LG	350.0	369.9
PLA/EGDE/5LG	351.7	370.1
PLA/PEGDE/1LG	348.9	369.1
PLA/PEGDE/3LG	351.8	371
PLA/PEGDE/5LG	342.2	364.5

**Table 3:** Thermal properties of PLA and PLA/lignin composites from DSC heat scan.

Sample	Tg (°C)	Tcc (°C)	Tm1 (°C)	Tm2 (°C)	χc
Neat PLA	59	112.8	—	165.6	3.1%
PLA/1LG	60.1	111.4	163.7	170	5.7%
PLA/3LG	59.5	110.9	163.4	169.9	8.6%
PLA/5LG	59.6	113.4	163.8	169.8	7.9%
PLA/EGDE/1LG	50.6	104.7	158.1	167.3	5.1%
PLA/EGDE/3LG	51.4	104.8	158.3	167.4	7.3%
PLA/EGDE/5LG	51.5	110	159.5	167.6	5.9%
PLA/PEGDE/1LG	50.1	100.4	—	167.4	4.6%
PLA/PEGDE/3LG	51.1	103.3	158.3	167.6	4.0%
PLA/PEGDE/5LG	52.2	104.1	158.6	167.9	6.8%

**Table 4:** O_2_ permeability of neat PLA and PLA/lignin composite films.

Sample	OTR (Barrer)	Reduction (%)
Neat PLA	0.218	0
PLA/1LG	0.106	51.4
PLA/3LG	0.125	42.7
PLA/5LG	0.110	49.5
PLA/EGDE/1LG	0.119	45.4
PLA/EGDE/3LG	0.114	47.7
PLA/EGDE/5LG	0.091	58.3
PLA/PEGDE/1LG	0.125	42.7
PLA/PEGDE/3LG	0.119	45.4
PLA/PEGDE/5LG	0.093	57.3

## Data Availability

Data sharing not applicable.

## References

[1] Rai P., Mehrotra S., Priya S., Gnansounou E., Sharma S.K. (2021). Recent advances in the sustainable design and applications of biodegradable polymers. Bioresour. Technol..

[2] Ajibade F.O., Adelodun B., Lasisi K.H., Fadare O.O., Ajibade T.F., Nwogwu N.A., Sulaymon I.D., Ugya A.Y., Wang H.C., Wang A. (2021). Environmental pollution and their socioeconomic impacts. Microbe Mediated Remediation of Environmental Contaminants.

[3] Wojnowska-Baryła I., Kulikowska D., Bernat K. (2020). Effect of bio-based products on waste management. Sustainability.

[4] Ruggero F., Gori R., Lubello C. (2019). Methodologies to assess biodegradation of bioplastics during aerobic composting and anaerobic digestion: A review. Waste Manag. Res..

[5] Swetha T.A., Bora A., Mohanrasu K., Balaji P., Raja R., Ponnuchamy K., Muthusamy G., Arun A. (2023). A comprehensive review on polylactic acid (PLA)–Synthesis, processing and application in food packaging. Int. J. Biol. Macromol..

[6] Grzebieniarz W., Biswas D., Roy S., Jamróz E. (2023). Advances in biopolymer-based multi-layer film preparations and food packaging applications. Food Packag. Shelf Life.

[7] Ekielski A., Żelaziński T., Mishra P.K., Skudlarski J. (2021). Properties of biocomposites produced with thermoplastic starch and digestate: Physicochemical and mechanical characteristics. Materials.

[8] Perera K.Y., Jaiswal A.K., Jaiswal S. (2023). Biopolymer-Based Sustainable Food Packaging Materials: Challenges, Solutions, and Applications. Foods.

[9] Wypij M., Trzcińska-Wencel J., Golińska P., Avila-Quezada G.D., Ingle A.P., Rai M. (2023). The strategic applications of natural polymer nanocomposites in food packaging and agriculture: Chances, challenges, and consumers’ perception. Front. Chem..

[10] Hartmann M. (1998). High molecular weight polylactic acid polymers. Biopolymers from Renewable Resources.

[11] Hu Y., Daoud W.A., Cheuk K.K.L., Lin C.S.K. (2016). Newly developed techniques on polycondensation, ring-opening polymerization and polymer modification: Focus on poly(lactic acid). Materials.

[12] Pohanka M. (2020). D-lactic acid as a metabolite: Toxicology, diagnosis, and detection. BioMed Res. Int..

[13] Jamshidian M., Tehrany E.A., Imran M., Akhtar M.J., Cleymand F., Desobry S. (2012). Structural, mechanical and barrier properties of active PLA–antioxidant films. J. Food Eng..

[14] Bhatia A., Gupta R.K., Bhattacharya S.N., Choi H. (2007). Compatibility of biodegradable poly (lactic acid)(PLA) and poly (butylene succinate)(PBS) blends for packaging application. Korea-Aust. Rheol. J..

[15] Xu T., Tang Z., Zhu J. (2012). Synthesis of polylactide-graft-glycidyl methacrylate graft copolymer and its application as a coupling agent in polylactide/bamboo flour biocomposites. J. Appl. Polym. Sci..

[16] Abdillahi H., Chabrat E., Rouilly A., Rigal L. (2013). Influence of citric acid on thermoplastic wheat flour/poly (lactic acid) blends. II. Barrier properties and water vapor sorption isotherms. Ind. Crops Prod..

[17] de França J.O.C., da Silva Valadares D., Paiva M.F., Dias S.C.L., Dias J.A. (2022). Polymers based on PLA from synthesis using D,L-lactic acid (or racemic lactide) and some biomedical applications: A short review. Polymers.

[18] Murariu M., Dubois P. (2016). PLA composites: From production to properties. Adv. Drug Deliv. Rev..

[19] McKeown P., Jones M.D. (2020). The chemical recycling of PLA: A review. Sustain. Chem..

[20] Trivedi A.K., Gupta M.K., Singh H. (2023). PLA based biocomposites for sustainable products: A review. Adv. Ind. Eng. Polym. Res..

[21] Claro P., Neto A., Bibbo A., Mattoso L., Bastos M., Marconcini J. (2016). Biodegradable blends with potential use in packaging: A comparison of PLA/chitosan and PLA/cellulose acetate films. J. Polym. Environ..

[22] Ostafinska A., Fortelny I., Nevoralova M., Hodan J., Kredatusova J., Slouf M. (2015). Synergistic effects in mechanical properties of PLA/PCL blends with optimized composition, processing, and morphology. RSC Adv..

[23] Arrieta M.P., Samper M.D., López J., Jiménez A. (2014). Combined effect of poly (hydroxybutyrate) and plasticizers on polylactic acid properties for film intended for food packaging. J. Polym. Environ..

[24] Bouakaz B.S., Pillin I., Habi A., Grohens Y. (2015). Synergy between fillers in organomontmorillonite/graphene–PLA nanocomposites. Appl. Clay Sci..

[25] Banu J.R., Kavitha S., Kannah R.Y., Devi T.P., Gunasekaran M., Kim S.-H., Kumar G. (2019). A review on biopolymer production via lignin valorization. Bioresour. Technol..

[26] Kumar A., Tumu V.R., Chowdhury S.R., SVS R.R. (2019). A green physical approach to compatibilize a bio-based poly (lactic acid)/lignin blend for better mechanical, thermal and degradation properties. Int. J. Biol. Macromol..

[27] Ou W.-X., Weng Y., Zeng J.-B., Li Y.-D. (2023). Fully biobased poly (lactic acid)/lignin composites compatibilized by epoxidized natural rubber. Int. J. Biol. Macromol..

[28] Hong S.-H., Park J.H., Kim O.Y., Hwang S.-H. (2021). Preparation of chemically modified lignin-reinforced PLA biocomposites and their 3D printing performance. Polymers.

[29] Park C.-W., Youe W.-J., Kim S.-J., Han S.-Y., Park J.-S., Lee E.-A., Kwon G.-J., Kim Y.-S., Kim N.-H., Lee S.-H. (2019). Effect of lignin plasticization on physico-mechanical properties of lignin/poly(lactic acid) composites. Polymers.

[30] Wasti S., Triggs E., Farag R., Auad M., Adhikari S., Bajwa D., Li M., Ragauskas A.J. (2021). Influence of plasticizers on thermal and mechanical properties of biocomposite filaments made from lignin and polylactic acid for 3D printing. Compos. Part B Eng..

[31] Yetiş F., Liu X., Sampson W.W., Gong R.H. (2020). Acetylation of lignin containing microfibrillated cellulose and its reinforcing effect for polylactic acid. Eur. Polym. J..

[32] Shi K., Liu G., Sun H., Weng Y. (2023). Polylactic Acid/Lignin Composites: A Review. Polymers.

[33] Yang W., Weng Y., Puglia D., Qi G., Dong W., Kenny J.M., Ma P. (2020). Poly(lactic acid)/lignin films with enhanced toughness and anti-oxidation performance for active food packaging. Int. J. Biol. Macromol..

[34] Iglesias-Montes M.L., Luzi F., Dominici F., Torre L., Manfredi L.B., Cyras V.P., Puglia D. (2021). Migration and degradation in composting environment of active polylactic acid bilayer nanocomposites films: Combined role of umbelliferone, lignin and cellulose nanostructures. Polymers.

[35] Xu J., Song R., Dai Y., Yang S., Li J., Wei R. (2019). Characterization of zinc oxide nanoparticles-epoxy resin composite and its antibacterial effects on spoilage bacteria derived from silvery pomfret (*Pampus argenteus*). Food Packag. Shelf Life.

[36] Wang X., Zhu J., Liu X., Zhang H.J., Zhu X. (2020). Novel gelatin-based eco-friendly adhesive with a hyperbranched cross-linked structure. Ind. Eng. Chem. Res..

[37] (2010). Standard Test Method for Wood Preservatives by Laboratory Soil-Block Cultures.

[38] (2008). Plastics—Film and sheeting—Determination of water vapour transmission rate—Part 3: Electrolytic detection sensor method.

[39] Narita J., Katagiri M., Tsuji H. (2013). Highly enhanced accelerating effect of melt-recrystallized stereocomplex crystallites on poly(L-lactic acid) crystallization: Effects of molecular weight of poly(D-lactic acid). Polym. Int..

[40] Spiridon I., Leluk K., Resmerita A.M., Darie R.N. (2015). Evaluation of PLA–lignin bioplastics properties before and after accelerated weathering. Compos. Part B Eng..

[41] Wang N., Zhang C., Weng Y. (2021). Enhancing gas barrier performance of polylactic acid/lignin composite films through cooperative effect of compatibilization and nucleation. J. Appl. Polym. Sci..

[42] Liao Y., Liu C., Coppola B., Barra G., Di Maio L., Incarnato L., Lafdi K. (2019). Effect of porosity and crystallinity on 3D printed PLA properties. Polymers.

[43] Courgneau C., Domenek S., Colomines G., Guinault A., Avérous L., Ducruet V. Effects of poly(lactic acid) formulation and crystallinity on barrier properties. Proceedings of the 8th World Congress of Chemical Engineering (WCCE8).

[44] Cocca M., Di Lorenzo M.L., Malinconico M., Frezza V. (2011). Influence of crystal polymorphism on mechanical and barrier properties of poly(L-lactic acid). Eur. Polym. J..

[45] Chieng B.W., Ibrahim N.A., Wan Yunus W.M.Z., Hussein M.Z., Then Y.Y., Loo Y.Y. (2014). Effects of graphene nanoplatelets and reduced graphene oxide on poly(lactic acid) and plasticized poly(lactic acid): A comparative study. Polymers.

[46] Kovalcik A., Pérez-Camargo R.A., Fürst C., Kucharczyk P., Müller A.J. (2017). Nucleating efficiency and thermal stability of industrial non-purified lignins and ultrafine talc in poly(lactic acid) (PLA). Polym. Degrad. Stab..

[47] Lotz B., Li G., Chen X., Puiggali J. (2017). Crystal polymorphism of polylactides and poly(Pro-*alt*-CO): The metastable beta and gamma phases. Formation of homochiral PLLA phases in the PLLA/PDLA blends. Polymer.

[48] Zhou J., Luo X., Lin X. (2006). Progress and prospect of the degradable foams of starch and lignin. Chem. Ind. Eng. Prog..

